# Evaluation of the InterRAI Early Years for Degree of Preterm Birth and Gross Motor Delay

**DOI:** 10.3389/fpsyg.2022.788290

**Published:** 2022-02-22

**Authors:** Jo Ann M. Iantosca, Shannon L. Stewart

**Affiliations:** ^1^School of Early Childhood Education, Seneca College, Toronto, ON, Canada; ^2^Faculty of Education, Western University, London, ON, Canada

**Keywords:** preterm, low birth weight, interRAI, early years, validation

## Abstract

**Background:**

The interRAI 0–3 Early Years was recently developed to support intervention efforts based on the needs of young children and their families. One aspect of child development assessed by the Early Years instrument are motor skills, which are integral for the maturity of cognition, language, social-emotional and other developmental outcomes. Gross motor development, however, is negatively impacted by pre-term birth and low birth weight. For the purpose of known-groups validation, an at-risk sample of preterm children using the interRAI 0–3 Early Years was included to examine correlates of preterm risk and the degree of gross motor delay.

**Methods:**

Participant data included children and families (*n* = 591) from 17 health agencies in Ontario, Canada. Data were collected as part of a pilot study using the full interRAI 0–3 Early Years assessment. Correlational analyses were used to determine relationships between prenatal risk and preterm birth and bivariate analyses examined successful and failed performance of at-risk children on gross motor items. A Kruskal-Wallis test was used to determine the mean difference in gross motor scores for children born at various weeks gestation.

**Results:**

Correlational analysis indicated that prenatal and perinatal factors such as maternal nicotine use during pregnancy did not have significant influence over gross motor achievement for the full sample, however, gross motor scores were lower for children born pre-term or low birth weight based on bivariate analysis. Gross motor scores decreased from 40 weeks’ gestation (mean rank = 310.77), to moderate to late preterm (mean rank = 258.96), and to very preterm (mean rank = 234.54), however extremely preterm (mean rank = 236.28) performed comparably to very preterm.

**Interpretation:**

The interRAI 0–3 was evaluated to determine its efficacy and report findings which confirm the literature regarding delay in gross motor performance for preterm children. Findings confirm that pre-term and low birth weight children are at greater risk for motor delay *via* the interRAI 0–3 Early Years gross motor domain.

## Introduction

Children who are born preterm (PT), or low birth weight (LBW) face additional barriers as compared to normal birth weight and full-term children, including risk of chronic developmental (i.e., motor, cognitive, communicative), behavioral, socio-emotional, and psychological difficulties. These children are also more likely to have a diagnosed neurodevelopmental or learning disability as compared to full-term children (Cheadle and Goosby, 2010; Shah et al., 2013; Månsson and Stjernqvist, 2014; Gladstone et al., 2015; Fevang et al., 2016; Johnson et al., 2016). When born LBW or PT, the neonate can be impacted by immediate medical complications such as respiratory distress or intraventricular hemorrhage, and future conditions of diabetes, heart disease and other health conditions (OECD, 2013). In concert, families undergo significant stress due to the additional challenges in financially, physically, and emotionally supporting their child (Cheadle and Goosby, 2010; Hodek et al., 2011; Gerstein and Poehlmann-Tynan, 2015). Preterm birth and low birthweight also impact the longitudinal health and well-being of children and their families, making this an expansive population serviced by hospitals and other treatment facilities in Canada (Lim et al., 2009; Treyvaud et al., 2014).

Children born prior to 37 weeks’ gestation are considered PT, and infants with a birthweight of under 5.5 pounds are identified as LBW regardless of gestational age (World Health Organization et al., 2012; OECD, 2013). Although infant mortality has decreased in many developed countries, the incidence of children born with low birth weight is increasing, with estimates in Canada at 6.3 percent, and late preterm births rising 20% from 1990 to 2006 in the United States (National Center for Health Statistics, 2009; OECD, 2013). Increasingly, more attention has been given to children born late preterm, between the gestational age of 34–36 weeks of pregnancy, due to recently observed disparities in health and developmental outcomes (Raju, 2006; National Center for Health Statistics, 2009; Woythaler et al., 2011; Johnson et al., 2015), however, extremely low birthweight (ELBW, < 1,000 g) or very preterm (VPT, 28–32 weeks) children are still at greatest risk (Mikkola et al., 2005; Cheadle and Goosby, 2010; Gladstone et al., 2015; Fevang et al., 2016). Internationally, the prevalence of preterm births falls around 10–11 percent, with LBW and PT more common in developing countries (Beck et al., 2010; Blencowe et al., 2012).

Preventable conditions such as poor maternal mental and physical health, maternal smoking or use of toxic substances, mothers’ age at birth, and inadequate prenatal care provide some explanation for the cause of this condition (Bandstra et al., 2010; World Health Organization et al., 2012; Finnegan, 2013; Bouras et al., 2015). A common maternal health complication is gestational diabetes during pregnancy. Type 2 diabetes as diagnosed at or before 26 weeks’ gestation was found to be a leading risk for the later diagnosis of Autism Spectrum Disorder (ASD), while controlling for several other common predictors such as maternal smoking, body mass index and socio-economic status (Xiang et al., 2015). Maternal age during pregnancy has also been found to predict low birth weight and preterm birth, in addition to elective caesarian surgery, and post-health outcomes for the mother (Oakley et al., 2016). Prenatal exposure to substances such as illicit drugs and alcohol, are responsible for health and developmental problems in childhood and adolescence and can lead to increased likelihood of preterm birth (Bandstra et al., 2010; Finnegan, 2013; O’Keeffe et al., 2014). Finally, maternal stress *in utero* is linked to low birth weight or preterm birth, however this evidence has not been conclusive when examining stress hormones (Nkansah-Amankra et al., 2010; Kramer et al., 2013; Romero-Gonzalez et al., 2018).

Non-maternal characteristics of preterm birth include being a product of multiple birth, and time spent in a neonatal intensive care unit (NICU). Many preterm or low birth weight children are likely to have spent time in a NICU, impacting the development of sensory systems and ultimately affecting later outcomes in language, cognition and motor areas (Subedi et al., 2017; Vandormael et al., 2019). In one study, preterm children were assessed at multiple time points from 9 months of age into kindergarten, and authors found that the extent of preterm birth as measured by gestational age no longer predicted child outcomes, but rather, the increased length of stay in NICU predicted milestone achievement more substantially (Subedi et al., 2017). Due to any number of maternal and non-maternal issues, children born preterm or low birth weight have broad deficits impacting their development.

Researchers have been examining the continued effects of PT and LBW, including a number of health and developmental issues that are present prior to and beyond kindergarten. Major areas of research revolve around the social competence and behavioral presentation of children born PT or LBW, as well as their cognitive development and academic performance in later life.

Children born PT and LBW display greater dysfunctional behavior, reduced social competence, and a wide range of psychosocial concerns as compared to their full term and normal-birth-weight peers (Jones et al., 2013; Fevang et al., 2016). In a meta-analysis of recent literature, authors found that young children born with severe levels of PT or LBW struggled with poor emotional regulation, social skills, and had more attentional problems as compared to full term children, which predicted future dysfunctional behavior into school age, regardless of cognitive performance (Arpi and Ferrari, 2013). LBW and preterm birth also lead to high levels of maternal stress and burdens in child-parent interactions, potentially impacting the behavioral outcomes of these children (Yates et al., 2010; Woythaler et al., 2011; Poulsen et al., 2013; Ritter et al., 2013; Gerstein and Poehlmann-Tynan, 2015; Fevang et al., 2016; Gerstein et al., 2017). Executive functioning is significantly correlated with childhood social competence, with impairments in executive function prevalent amongst PT and LBW children, particularly childhood inhibitory control (Jones et al., 2013; Ritter et al., 2013; Alduncin et al., 2014).

Children with severe low birth weight and very preterm birth who demonstrate an early delay in executive functioning, may also display cognitive impairment beyond adolescence and into adulthood (Ritter et al., 2013; Eryigit Madzwamuse et al., 2015). It has also been observed that late and moderately preterm children demonstrate significant delays in cognitive function as well (Johnson et al., 2015). In the early years, low birth weight and preterm children demonstrate significantly lower motor, communication and cognitive skills as compared to full-term children (Månsson and Stjernqvist, 2014; Peyton et al., 2018). Even the early abilities of infants to use gestures and other forms of receptive language is affected by these vulnerabilities, which tends to create conditions for future identification of learning disabilities in the school setting (Barre et al., 2011; Johnson et al., 2016; Stolt et al., 2016). Likewise, childhood motor development, often seen as partly responsible for early cognitive function, is negatively impacted by pre-term birth or low birth weight, regardless of diagnosis of physical disability (Van Hus et al., 2013; Sansavini et al., 2014). It is this coordinated process of tuning the gross and fine motor systems that prepares children for more complex tasks in later childhood. Motor skills are crucial in determining independence of children on such tasks as dressing, feeding, hygiene-related activities, as well as on oral and written academic tasks in school settings (Houwen et al., 2016; MacDonald et al., 2016). Children across all levels of severity are at risk for achieving lower IQ scores, more likely to receive placement in special education, as well as decreased academic scores across reading, writing and mathematics as compared to normal-birthweight children (Poulsen et al., 2013; Basten et al., 2015). Even while controlling for the effects of family socio-economic status, for instance, the poor educational performance of preterm children can lead to future decreases in educational attainment later in life, and similarly, less well-paying positions of employment (Basten et al., 2015).

The early intervention literature pertaining to preterm and low birth weight children is scarce and often immaterial (see Johnson (2009), Evans et al. (2017)), however, the early effects of LBW and PT birth on infant and toddler development should be explored in order to enhance early intervention efforts.

With early intervention, it is also crucial to use strong measures of infant and toddler development that pertain to the unique needs of low birthweight and preterm children across specific developmental domains. Few recent studies have evaluated currently used infant and toddler assessments of developmental milestones (see Greene et al. (2012), Sansavini et al. (2014), Lefebvre et al. (2016), Agarwal et al. (2017)). Commonly administered instruments have also been criticized for inaccurate cut offs amongst very preterm or low birth weight children (such as by overestimating motor impairment), as well as unexplained variance in predicting future motor function and classification instability over time (see Luttikhuizen dos Santos et al. (2013), Lobo et al. (2014), Duncan et al. (2015)). In a recent meta-analysis investigating the predictive capacity of future cognitive outcomes for preterm and low birth weight children, common early childhood assessments such as the Bayley Scales of Infant Development, had greater specificity overall, but sensitivity was typically lower when examining future outcomes (Wong et al., 2016). Wong et al. (2016) recommended that test developers examine more closely the predictive accuracy of their screens, and link to consistent follow up assessment in order to increase the odds of detecting later delay. However, others have discovered findings that are strongly predictive of determining developmental delay amongst preterm and low birth weight infants (Agarwal et al., 2017). The accuracy of tests is also important to help determine resource allocation. The resources needed to service this population in Canada ranges based on birthweight and preterm birth, with the cost growing substantially higher than for children born full term and normal birthweight (Lim et al., 2009). For instance, those who are born in the range of 1,000–1,499 grams, cost an average of $50,000 as newborns, and for those born preterm at any gestational age, costing $9,233 and up to $84,235 when extremely preterm (Lim et al., 2009). Thus, for the purposes of early intervention, it is crucial to determine the immediate consequences of preterm and low birth weight newborns by evaluating commonly administered screening and assessment tools for this population.

interRAI is a non-profit conglomerate of researchers from around the world, who develop assessment systems to target the needs of individuals across the lifespan. The child and youth suite of assessments includes the interRAI 0–3 Early Years (Stewart et al., 2017), which has been developed to identify the overall developmental needs of young children between 0 and 47 months of age, as well as their family. The interRAI 0–3 captures more than 650 items that seek insight on ecological risk factors, family dynamics, medical and mental health information, as well as all areas of early development. It provides information specific to early identification and intervention (e.g., preterm birth, low birthweight, caregiver distress, emotion dysregulation). Items include multilevel assessment of frequency and intensity (e.g., *Present recently but not exhibited in last 3 nights/days*), performance and capacity of tasks (e.g., *Extensive assistance- help throughout task, but performs 50% or more of task on own*), and age-related items indicating presence or non-presence of developmental achievement (e.g., *Grasping- picks up tiny objects with fingertips (e.g., food crumbs, peas)*). Items are carried forward from the child and youth suite of instruments as applicable, and new items undergo a rigorous approval process by an Instrument and Systems Development (ISD) committee before pilot and publication. The interRAI 0–3 Early Years is currently in pilot testing, evaluating the efficacy of its items and scales before final approval for submission to publication can be given.

This newly established instrument, however, has yet to explore the development of preterm children under the age of four in the motor domain. In the present study, data from the interRAI 0–3 Early Years was used to explore the motor findings of children at risk due to issues such as preterm birth, or low birthweight, seeking to understand how children between 6 and 47 months perform on gross motor outcomes based on extent of prematurity and other risk factors.

## Materials and Methods

### Sample

Participants completed the interRAI 0–3 Early Years as a part of a pilot study across 17 sites which provide developmental or mental health services in Ontario, Canada. This convenience sample included 591 children between the ages of 6–47 months of age (*M* = 31.6, *SD* = 12.71), with a majority of male participants (62.4%; see Table 1). As many as 20.3% (*n* = 120) identified as preterm (< 37 weeks) by the assessor during intake. This item utilized a binary response item to record for preterm birth based on caregiver response or an examination of medical documentation. More children were identified as preterm based on a separate item on the 0–3 Early Years, indicated by the number of weeks premature. This item indicated that 24.3% of children had a gestational age under 39 weeks. The majority were considered moderate to late preterm (16.4%), and only 11.2% of the sample was considered low birthweight. Much of the sample had been placed in some level of neonatal care after birth (43.1%), and 28.3% of mothers had health complications during the pregnancy or delivery. The most common health complications included gestational diabetes, hypertensive disorders, and fetal distress.

**TABLE 1:** Characteristics of interRAI 0–3 participants 6–47 months (*n* = 591).

Variables	Frequency (%)	Mean	*SD*
Age at assessment		31.6	12.7
**Sex**			
Male	369 (62.4)		
Female	222 (37.5)		
**Preterm birth**			
Yes	120 (20.3)		
No	471 (79.7)		
**Levels of prematurity**			
Extremely preterm (≤ 28 weeks)	16 (2.7)		
Very preterm (≤ 32 weeks)	37 (6.3)		
Moderate/late preterm (≤ 39 weeks)	91 (15.4)		
40 Weeks’ gestation (≥ 40 weeks)	447 (75.6)		
Low birth weight*			
Yes	61 (10.3)		
No	482 (81.5)		
Neonatal intensive care*			
Yes	222 (37.5)		
No	293 (49.6)		
Maternal health problems during pregnancy or delivery*			
Yes	142 (24.0)		
No	360 (60.9)		
Maternal nicotine use during pregnancy*			
Yes	83 (14.0)		
No	437 (73.9)		
Maternal alcohol use during pregnancy*			
Yes	26 (4.3)		
No	531 (89.8)		

*Missing data was unreported by assessors or not inputted into beta software.

### Measure

The interRAI 0–3 Early Years is a needs-based integrated assessment-to-intervention system that amalgamates social, psychiatric, medical, functional, psychological, and environmental constructs to evaluate and intervene based on the needs of young children and their families. Upon intake within child and family agencies across Ontario, assessors who received training on the interRAI 0–3 began to collect data with the child and family using the above measures. The interRAI 0–3 training included an overview of the form, manual, coding procedures, and practice using case studies. Pediatricians, psychiatrists, psychologists, infant therapists, early childhood educators, child and youth workers, child life specialists, and early intervention teams administered the interRAI 0–3. Assessors were required to have a diploma or degree in early child development, at least 2 years of work experience with young children, and have received the comprehensive *interRAI 0–3* 2-day assessor training program. The *interRAI 0–3* uses a clinician-rated semi-structured interview format and requires approximately 45–90 min to complete depending on case complexity, age of the child and assessor experience. Initial assessments may require additional time due to the novelty of the case. Clinicians were given explicit instruction to use information from multiple sources such as medical documentation where approved, as well as information from the caregivers, extended family, childcare providers or other individuals relevant to the context of the family. If clinicians felt that there was incongruent information based on the report from multiple sources, clinicians were asked to make observational judgments to validate their decisions where possible. The focus of the interRAI 0–3 Early Years measure was the Gross Motor domain, which is a multi-item scale that assesses the developmental milestones achieved in multiple age intervals including early mobility in infancy and the progression of climbing and running as the child matures in age. The presence of these milestones is determined using a 2-point coding structure (*0* = *No* to *1* = *Yes*), which is summed to provide a composite score based on the age range completed.

To test for gross motor performance, corrected age was used for children above 24 months by subtracting weeks premature by chronological age, indicating the child’s corrected age at assessment. For children under 24 months, gross motor outcomes were not adjusted based on corrected age.

### Statistical Analysis

The current study initially sought to examine the correlations between risk items (i.e., premature birth, low birthweight, neonatal intensive care, maternal nicotine and alcohol use, and maternal health problems) and performance on gross motor milestones as a means to discover convergence between risk items and associations with gross motor performance. Next, bivariate associations were used to discover the successful and failed performance of *at risk* and *no risk* children on the interRAI 0–3 gross motor domain. Initially, contingency tables and chi square were calculated for predictors of developmental outcomes for premature children based on the literature. Proposed variables that contribute to poor developmental outcomes included maternal age, premature birth, birthweight, maternal health problems, stay in NICU, as well as maternal nicotine and alcohol use. Though important to this research, variables not included in the analysis were *assistive reproductive technology used to achieve pregnancy*, and *child is a product of multiple birth*, as this subsample of participants was not substantive. This dataset includes some missing data, which was unreported or not inputted into beta software by assessors.

Finally, an independent-samples Kruskal-Wallis test was conducted to examine the gross motor outcomes of children born extremely preterm (at or below 28 weeks’ gestation), very preterm (at or below 32 weeks’ gestation), moderate to late preterm (33–39 weeks’ gestation) and at 40 weeks’ gestation or having no reported preterm birth. A non-parametric test was chosen as a test of normality revealed that homogeneity of variances could not be assumed. Box-plots were used to determine differences in scores across levels of prematurity, a means test was carried out and *post hoc* tests were used to determine levels of significance among gross motor scores between categories.

## Results

Initially, Pearson-product moment correlations were run to seek evidence between performance on gross motor items and variables that place children at risk of poor performance (see Table 2). Items from the interRAI 0–3 that were used included preterm birth and low birthweight, stay in a NICU, maternal health problems during pregnancy and maternal nicotine use during pregnancy. Interestingly, the findings showed significant negative correlations between performance on gross motor and all risk-oriented items except for nicotine use during pregnancy, however, the strength of relationship between other items was weak. While the direction of the relationship is not clear, either an improvement in performance on gross motor leads to decreased risk, or an increase in risk leads to poor performance on gross motor items. Correlations between risk-items were also sought, indicating convergence between constructs that are commonly known to load together. Children with any known risk, such as preterm birth, was found to relate to other risk factors such as receipt of neonatal intensive care.

**TABLE 2:** Correlation matrix between gross motor performance and risk factors for development.

Items		Low birthweight	Stay in neonatal intensive care unit	Maternal health problems during pregnancy	Maternal nicotine use during pregnancy	Maternal alcohol use during pregnancy	Gross motor performance (pass/fail)
Preterm birth	Pearson correlation	0.389**	0.496**	0.283**	0.013	–0.025	–0.154**
	Significance (2-tailed)	0.000	0.000	0.000	0.766	0.562	0.000
	N	591	561	545	564	558	591
Low birthweight	Pearson correlation		0.300**	0.096*	–0.042	0.060	–0.110*
	Significance (2-tailed)		0.000	0.029	0.339	0.169	0.000
	N		528	516	533	528	543
Stay in neonatal intensive care unit	Pearson correlation			0.235**	0.031	0.032	–0.200**
	Significance (2-tailed)			0.000	0.483	0.466	0.000
	N			510	525	521	515
Maternal health problems during pregnancy or delivery	Pearson correlation				–0.021	0.004	–0.108*
	Significance (2-tailed)				0.635	0.935	0.000
	N				521	521	502
Maternal nicotine use during pregnancy	Pearson correlation					0.296**	0.043
	Significance (2-tailed)					0.000	0.331
	N					548	520
Maternal alcohol use during pregnancy	Pearson correlation						0.135**
	Significance (2-tailed)						0.002
	N						514

*Denotes statistical significance at the 0.05 level. **Denotes statistical significance at the 0.01 level.

Using items from the interRAI 0–3, common predictive risk factors were chosen to explore associations with developmental outcomes on the gross motor domain as a stronger measure of relationships between variables (see Table 3). The findings suggest that children with no identified risks were more likely to achieve gross motor milestones at a higher rate than those with identified risk factors. The gross motor findings indicated that within the at-risk group, most children identified as being preterm, low birthweight or having other risks for developmental delay were found to succeed or fail milestones nearly equally. The risk estimates for each variable, however, show that passing as compared to failing gross motor milestones for preterm birth, low birthweight, maternal health issues during pregnancy, or being in neonatal intensive care does not increase the risk estimate to above 1. Conversely, maternal nicotine use (1.27), and alcohol use during pregnancy (5.51) did lead to an increased risk estimate, with the group that failed gross motor milestones (1.16; 3.62), respectively, showing a risk estimate above 1.

**TABLE 3:** Bivariate association between achievement of gross motor milestones and predictors for children 6–47 months (*n* = 591).

Variables	Achievement of gross		Chi-square (sig.)
	motor milestones		
	Yes	No	
Preterm (<37 weeks)			0.000 (0.001)
Yes	55 (45.8)	65 (54.2)	
No	304 (64.5)	167 (35.5)	
Low birth weight (<1,500 g)*			
Yes	28 (45.9)	33 (54.1)	0.011 (0.001)
No	303 (62.9)	179 (37.1)	
Neonatal intensive care*			0.000 (0.001)
Yes	110 (49.5)	112 (50.5)	
No	203 (69.3)	90 (30.7)	
Maternal health problems during pregnancy or delivery*			0.015 (0.001)
Yes	72 (50.7)	70 (49.3)	
No	225 (62.5)	135 (37.5)	
Maternal nicotine use during pregnancy*			0.330
Yes	53 (63.9)	30 (36.1)	
No	254 (58.1)	183 (41.9)	
Maternal alcohol use during pregnancy*			0.002 (0.001)
Yes	23 (88.5)	3 (11.6)	
No	284 (58.2)	204 (41.8)	

*Missing data was unreported by assessors or not inputted into beta software.

Initially, the number of weeks a child was born prematurely was converted into categories of extremely premature, very premature, moderate to late premature and 40 weeks’ gestation. Children who were at least 2 years of age and were born prematurely, would be asked to perform a set of items within their corrected age range. For children under 24 months, gross motor items were not adjusted based on corrected age. These variables were then examined for normal distribution according to the Shapiro-Wilk’s test of normality. The results indicate that although the very preterm category was considered normally distributed, all other levels of prematurity did not meet the normality assumption.

Given the low and unequal sample sizes within each category, a non-parametric test was selected in order to reduce type I error (Kruskal and Wallis, 1952). An independent-samples Kuskal-Wallis Test was used, and initial examination of the boxplot indicated that distributions of gross motor scores were different for each level of premature birth. The distributions of gross motor scores were significantly different across categories of prematurity [*H*(3) = 15.520, *p* = 0.001], thus the null hypothesis was rejected. Gross motor scores decreased from 40 weeks’ gestation (mean rank = 310.77), to moderate to late preterm (mean rank = 258.96), and to very preterm (mean rank = 234.54), however extremely preterm (mean rank = 236.28) performed comparably to very preterm.

Given the level of significance, pairwise comparisons using Bonferroni correction were executed. Accepting statistical significance based on adjusted *p*-values at the *p* < 0.05 level revealed differences between gross motor scores for two categories. *Post hoc* analysis showed statistical significance between gross motor scores for very preterm birth and 40 weeks’ gestation (*p* = 0.04), and between moderate to late preterm and 40 weeks’ gestation (*p* = 0.04), but not between other groups.

## Discussion and Conclusion

The present study examined relationships between perinatal and prenatal risk for gross motor delay, including preterm birth and low birthweight, stay in NICU, maternal health problems as well as nicotine and alcohol use during pregnancy. Next, the mean gross motor scores of children were compared based on levels of preterm birth.

Initially, a correlation matrix was generated to examine the relationship between risk-items on the interRAI 0–3 and their association with pass/fail performance of gross motor milestones. The results indicated that items such as preterm birth and low birthweight, time in a NICU, and maternal health problems during pregnancy or delivery are all positively and significantly correlated with one another, however, maternal nicotine and alcohol use were not correlated with these other risk factors, rather correlated with one another. An increase in any one of the correlated risk factors indicate that the others will also linearly increase. This is an important finding, as it shows that multiple interRAI 0–3 items that link to preterm birth show convergence, however, this also increases the likelihood of multicollinearity in any logistic model. Additionally, these items all show a negative relationship with pass/fail outcomes from the gross motor domain, which is a common finding in the literature for preterm children. Conversely, alcohol use during pregnancy showed a positive statistically significant relationship, which is likely due to limited sample size (*n* = 26). Moreover, nicotine use was also scarcely reported amongst maternal participants (*n* = 83), finding no association to PT, or LBW. Additionally, the relationship between poor performance on gross motor outcomes was expected to be stronger for the at-risk population given the literature which shows that prenatal and perinatal factors have significant influence over gross motor achievement (Ghassabian et al., 2016a,b; Yaari et al., 2018). The present study found that the strength of correlations with gross motor outcomes ranged between –0.108 for maternal health problems during pregnancy and –0.200 for stay in a NICU. Finally, the risk estimate seemed to be highest for variables pertaining to alcohol and nicotine use, more than other perinatal and prenatal factors.

Of the risk factors discussed in this study, of particular interest was the necessity of neonatal intensive care. Much of the current literature shows that children born preterm require care by specialists in a NICU, and that a longer period of time spent in this type of care forecasts poorer developmental outcomes (Subedi et al., 2017). Staying in a NICU is also hypothesized to impact the infant beyond the effects of their prematurity or low birthweight by having increased medical interventions and reducing holding behavior (Pineda et al., 2018). An increase in holding the child leads to stronger tuning of the reflexes based on parent interventions (Pineda et al., 2018). There is evidence to suggest that neuromuscular development can be delayed due to length of stay in a NICU (Zuccarini et al., 2016), thus future research should further investigate this relationship using data from the interRAI 0–3 Early Years instrument.

The interRAI 0–3 adjusts for prematurity within all developmental domains for children under 24 months, which also may be responsible for the weak correlation with gross motor performance. Several assessments that measure child development correct for age by subtracting the number of weeks premature, by the child’s chronological age (see Bayley (2006), Bricker and Squires (2009)). We employed the same process to ensure that we capture accurately, the gross motor development of preterm children, as they are still biologically maturing. However, this has been criticized for underserving populations of children still considered at-risk for delay, noting that intervention services may be offered to less children who could still benefit from access (Yaari et al., 2018). Thus, it has been recommended that chronological and corrected age be considered for intervention purposes (Yaari et al., 2018). Future research using the interRAI 0–3 should examine participants scores within their age range without correcting for prematurity to find any measurable differences.

Bivariate associations with risk factors including preterm birth, low birthweight, time spent in a NICU and maternal health problems during pregnancy. These risk factors were found to be associated with higher risk of failure on gross motor domain items from the interRAI 0–3 by comparing at-risk children to the rest of the study population. For instance, 45.8% of children born preterm (< 37 weeks’ gestation), achieved motor outcomes as compared to 64.5% of children who were not born preterm. Similarly, children born with low birthweight achieved gross motor outcomes 45.9% of the time, with 62.9% of full-term children achieving milestones for their corrected age. This further reflects findings in the literature that suggest children who are considered preterm or low birthweight function below full term peers on motor outcomes (Sansavini et al., 2014; Lean et al., 2018; Yaari et al., 2018).

Within the group of preterm children in this study, more participants were likely to fail motor milestones. Specifically, of the children born preterm, 45.8% were able to achieve gross motor milestones, and 54.2% did not, and nearly identical findings for were discovered for the passing (45.9%) and failing (54.1%) low birthweight group. Yet, amongst the full-term cohort 64.5% of children achieved gross motor milestones for their age, and only 35.5% failed such milestones. Studies have found poorer results in very preterm and low birthweight children across all developmental domains (Lean et al., 2018; Yaari et al., 2018), thus future research should investigate associations between prenatal and perinatal risk factors using the extent of preterm birth. It may be that for children born pre-term, more immediate intervention services were given, leading to an indiscriminate difference between the participants who achieved or did not achieve particular milestones. Another important consideration pertains to the male predominance in this study (62.4%). Some authors suggest that evaluation of motor outcomes should include age and sex-specific assessment of motor skills during the early years given differences in fundamental motor skills (Kokštejn et al., 2017).

Lastly, the Kruskal-Wallis test was used to determine the mean difference in gross motor scores for children considered 40 weeks’ gestation, moderate to late preterm, very preterm and extremely preterm. Distributions amongst the groups varied at a statistically significant level, [*H*(3) = 15.520, *p* = 0.001], indicating that level of preterm birth effects the gross motor abilities of children, based on corrected age. The mean rank of 40 weeks’ gestation was highest, then moderate to late preterm, and very preterm, however, extremely preterm children performed slightly better, but not statistically superior to the very preterm category. Research suggests that the most at-risk groups (i.e., very preterm) tend to do most poorly on functional assessments, finding a reduced effect with children who are less severe (Schonhaut et al., 2013). It is posited that the small number of participants in the extremely preterm group (*n* = 16) were not sufficient to capture changes in the distribution. The only groups that were statistically significantly different in their achievement of gross motor milestones were the moderate to late preterm and the very preterm groups as compared to children considered 40 week’s gestation.

It must also be considered a limitation that the moderate to late preterm week’s gestation included cases of children between 33 and 39 weeks as opposed to 32–37 weeks in order to reduce case overlap and capture all children born before 40 weeks. In future, the moderate to late preterm group could be parsed out into early term and late preterm as sample size increases. The sample size of each group should be considered a limitation to interpretation of these findings. With an increased sample size, it would be interesting to examine preterm gross motor scores in infants as compared to older children in our sample, as there are early neuromuscular differences which lead to poor object manipulation at 6 months, and later motor difficulties in children at the age of 2 years (Zuccarini et al., 2016; Allotey et al., 2018).

The present study findings confirm that very preterm children perform poorly on gross motor outcomes as compared to full-term children, however, that late and moderate preterm birth are still suggestive of concern. Recent studies have been done to explore late preterm children, noticing significant differences in achievement across a broad range of milestones both early in childhood and later into school-age (Raju, 2006; National Center for Health Statistics, 2009; Woythaler et al., 2011; Johnson et al., 2015). The findings from this study reflect much of what is found in the literature and confirm the presence of concern for this population using data collected from interRAI 0–3. This helps to substantiate the use of the interRAI 0–3 as an instrument that accounts for levels of prematurity and prenatal and perinatal risk. Further research should explore predictive models based on maternal and post-term risk in order to replicate past studies and confirm the use of the interRAI 0–3 as predicting poorer developmental outcomes for this population. Future work should also consist of measuring the impact of preterm birth on different age cohorts in order to explore the longitudinal effects on gross motor development. Preterm birth and skill development in domains such as language, executive function and social-emotional areas should also be explored in order to replicate findings on preterm performance.

Following this, it would be interesting to explore different age groupings to see what is predictive for individual age ranges. This has been done in other research to counter the issue of developmental change, and more closely examine psychometric properties that appear to improve with the age at assessment (Schonhaut et al., 2013). The study population used for analysis also amalgamated new intake cases and those that may have been in a clinical program receiving early intervention. These cases could not be separated because this pilot study was the first of its kind to evaluate the interRAI 0–3, thus all cases in the database were considered an initial assessment. Future work will have the capability to separate first assessment from routine or discharge assessments. Finally, children who were considered preterm or low birthweight may have experienced other medical comorbidities or multiple diagnoses that impacted the association with these items. With increased data collection efforts, supplementary research into the role that comorbid diagnoses have on the preterm or low birthweight population could expand the impact of the interRAI 0–3.

Children who are preterm and low birthweight have been found to exhibit more delayed developmental trajectories than child who are born full-term and normal birthweight. With the incidence of low birth weight and late preterm birth rising, increased emphasis should be placed on investigating this vulnerable population. The interRAI 0–3 was examined for associations between risk factors for delay and levels of preterm birth on gross motor outcomes, which was an integral part of test development efforts.

## Data Availability Statement

The raw data supporting the conclusions of this article will be made available by the authors, without undue reservation.

## Ethics Statement

This study involved human participants and was approved by the University of Western Ontario Research Ethics Board (REB # 108024). The patients/participants provided their written informed consent to participate in this study.

## Author Contributions

JI composed the literature review, developed the analytical strategy, and performed the statistical analysis. SS provided intellectual direction, provided critical feedback to the manuscript, and was involved in the writing and reviewing of the final manuscript. Both authors contributed to the article and approved the submitted version.

## Conflict of Interest

The authors declare that the research was conducted in the absence of any commercial or financial relationships that could be construed as a potential conflict of interest.

## Publisher’s Note

All claims expressed in this article are solely those of the authors and do not necessarily represent those of their affiliated organizations, or those of the publisher, the editors and the reviewers. Any product that may be evaluated in this article, or claim that may be made by its manufacturer, is not guaranteed or endorsed by the publisher.
